# Capillary leak syndrome due to snakebite in the Amazon: case report

**DOI:** 10.17843/rpmesp.2024.414.13614

**Published:** 2024-10-25

**Authors:** Edgar A. Ramírez-García, Arley Perez-Mori, Mónica Mori-Coral, Maria Jose V. Canchanya-Olimar, Juan C. Celis-Salinas, Martín Casasapia-Morales

**Affiliations:** 1 Universidad Nacional de la Amazonia Peruana, Loreto, Peru. Universidad Nacional de la Amazonía Peruana Universidad Nacional de la Amazonia Peruana Loreto Peru; 2 Regional Hospital of Loreto, Loreto, Peru. ">Regional Hospital of Loreto Loreto Peru; 3 Scientific Society of Medical Students of the Peruvian Amazon, Loreto, Peru. Scientific Society of Medical Students of the Peruvian Amazon Loreto Peru

**Keywords:** Child, Snake Bites, Capillary Leak Syndrome

## Abstract

Capillary leak syndrome is a unique complication characterized by extravasation of liquids in the interstitial space due to protein loss caused by snakebite envenoming. We describe the case of a 12-year-old boy from the district of Napo in the city of Iquitos in the Peruvian Amazon, who had edema and increased face volume due to the bite of a snake of the Bothrops genus in the lateral aspect of the right leg; he was the hospitalized and diagnosed with severe ophidism complicated with face edema. The patient received eight vials of antivenin, antibiotics and analgesics. Finally, the patient was discharged from the hospital after eight days of hospitalization, with favorable evolution and recovery.

## INTRODUCTION

Snakebite envenoming was classified by the World Health Organization as one of the most important causes of mortality worldwide, the most affected regions being Southeast Asia, sub-Saharan Africa and South Asia ^1^^,^^2^. Globally, between 4.5 and 5.4 million cases of snakebites are reported each year, some 400,000 affected people are permanently disabled, and reported deaths range from 81,000 to 138,000 ^3^^,^^4^. In India, about 49,000 rural people die each year from snakebite envenoming ^5^. During 2023, a total of 461 cases of ophidism were reported in Peru, with Loreto (134), San Martín (96) and Ucayali (58) being the departments with the highest number of reports at the national level. The most affected age groups were adults with 43.4% and males with 67.3% ^6^. Recent estimates show that venomous snake bites cause multiple disabilities in those affected, mostly adults and young people from developing countries with inadequate access to health services, and that an even greater number of people are left with permanent physical and psychological sequelae ^7^^,^^8^.

Capillary leak syndrome is a unique complication caused by bites of snakes of the Viperidae family with a mortality rate of 58%, characterized by swelling of the parotid glands, chemosis, periorbital edema together with hypotension, hypoalbuminemia and hemoconcentration due to fluid extravasation, it is a fatal complication of variable etiology and of very low incidence ^9^. We present a probable case of Capillary Leakage Syndrome identified in the Peruvian Amazon, which contributes to make visible the importance of timely diagnosis and treatment for an adequate evolution of the disease, as well as to show an infrequent manifestation of the disease.

## CASE REPORT

**Figure 1:**
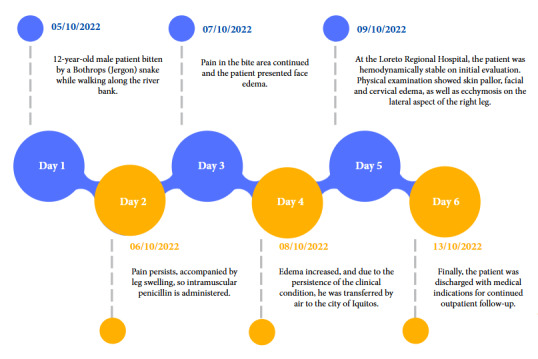
Timeline of the disease and evolution of patient with Bothrops bite

**Figure 2:**
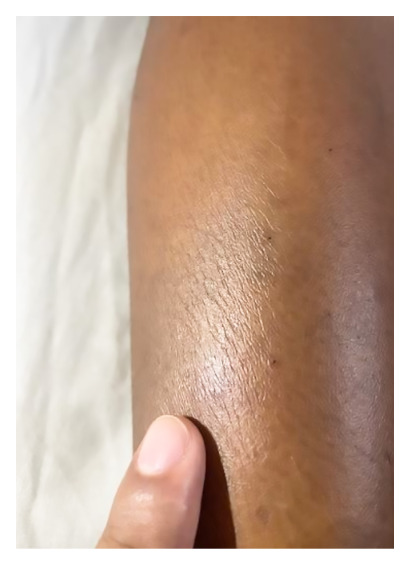
Ecchymosis on the lateral aspect of the right leg due to Bothrops bite.

**Figure 3:**
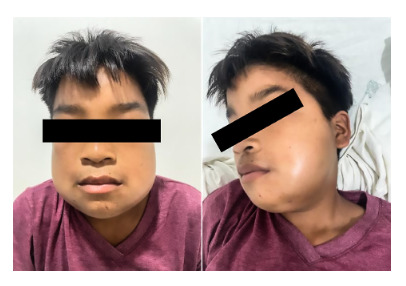
Capillary leak syndrome due to severe ophidism manifested by edema and swelling of the parotid glands on the face.

We present the case of a 12-year-old male patient from the community of Santa Clotilde on the Napo River located 1113 km from the city of Iquitos in the district of Napo, Maynas Province, Loreto region. The patient had no relevant infectious history, on day one (05/10/2022) he was bitten by a Bothrops (*Jergón*) snake while walking along the banks of the Napo River. According to the mother, the snake was approximately 50 cm long, with a triangular head, brown in color, with dark spots and a yellow tail. Three hours after the bite, he developed intense pain and bleeding in the area of the bite, lateral side of the right leg, for which he received home remedies (a glass with water and salt and a second glass with water and sugar). The next day (06/10/2022), the pain persisted, accompanied by inflammation in the leg, so he received intramuscular penicillin. On the third day (07/10/2022), the pain persisted in the area of the bite and the patient presented edema in the face, for which reason he was transferred to a health center where he was administered four vials of antivenom serum prepared by the Peruvian National Health Institute, dexamethasone and chlorphenamine. On the fourth day (08/10/2022) he developed increased edema, and due to persistence of the clinical picture he was transferred by air to the city of Iquitos, arriving at the Regional Hospital of Loreto on the fifth day (09/10/2022), during the evaluation the patient was hemodynamically stable, with vital signs within normal ranges (Figure 1). Physical examination showed cutaneous pallor, facial and cervical edema, as well as ecchymosis on the lateral aspect of the right leg (Figure 2). The parotid glands on the face were markedly swollen, suggesting a probable complication secondary to Bothrops envenoming (Figure 3).

The initial hemogram revealed mild anemia with a hemoglobin level of 9.9 g/dL (normal value ≥ 12.0 g/dL). Other complementary studies, including the coagulation profile, were found to be within normal parameters. He was treated with intravenous hydration of 0.9% sodium chloride and an antibiotic regimen with ceftriaxone (1.5 g every 12 hours) and clindamycin (385 mg every 8 hours), supplemented with four vials of antivenom serum (National Institute of Health) and metamizole (950 mg) for pain control.

After 7 days of treatment, the patient showed significant improvement, remaining hemodynamically stable with a notable decrease in edema and pain. Finally, he was discharged (10/13/2022) with medical indications to continue outpatient follow-up.

## DISCUSSION

The present case is of importance for medical practice in areas where bites by snakes of the Bothrops genus are frequent, such as the Peruvian Amazon. This type of envenomation causes a complex clinical picture that can present both local and systemic manifestations. This is the first reported case in the Peruvian Amazon of a probable capillary leak syndrome associated with ophidism. Clinical findings, such as facial edema, suggest the presence of this syndrome, a rare complication found in envenomation by snakes of the Viperidae family, which produces hemodynamic alterations characterized by myalgias, excessive thirst, swelling of the parotid glands, conjunctival chemosis and hypotension. A crucial aspect in the discussion of this case is the possible presentation of capillary leak syndrome. This disorder, although uncommon, is a serious complication in cases of snake envenomation. However, medical literature has documented most cases in studies from India, and its occurrence in South America is rarely reported. Capillary leak syndrome, also known as Clarkson’s disease, involves an increase in capillary permeability caused by elevated hydrostatic pressure in the capillaries, which can lead to cardiac and renal failure as well as hepatic venous obstruction ^10^^-^^12^.

Capillary leak syndrome is characterized by the presence of bilateral parotid swelling, chemosis and periorbital edema after a snakebite from the second or third day, the accumulation of fluid in the face has been termed “snakehead appearance” due to swelling of both parotid glands ^13^. Envenomation by Russell’s viper (Veperidae Family) causes vascular toxicity and hemorrhage, a variation that has also been found can lead to capillary leak syndrome causing widespread edema and massive plasma extravasation, a condition caused by profoundly increased vascular permeability, indicating that venom of the genus Viperidae is associated with this potentially fatal complication ^14^.

Diagnosis can be stablished by the presence of increased capillary permeability due to protein loss in the interstitial space with the appearance of fatigue, dizziness, edema, hemoconcentration and hypoalbuminemia. After a few days, permeability improves spontaneously and vital functions stabilize. Fluid management is an essential part of treatment because hypovolemia and hypotension can worsen the condition leading to multiorgan failure ^15^. However, the child presented inflammatory markers within normal ranges without protein loss and was managed with timely hydration, achieving improvement.

In conclusion, although the inflammatory markers were within normal ranges, features of severe ophidism with capillary leak syndrome were found. The main limitation of this case is the lack of diagnostic confirmation of capillary leak syndrome. However, the absence of fever, decreased hemoglobin without evidence of hemorrhage, and low leukocyte counts showed no association with other diseases. Clinical findings of edema from admission along with a bite mark with ecchymosis on the right leg characterized envenomation by snake of the genus Bothrops (Family Viperidae) causing the probable capillary leak syndrome. The child received eight vials of antivenom serum, four were administered at the referral center and four when he arrived at the hospital. After a few days of hospitalization, the patient recovered and was discharged on the eighth day of hospitalization.
