# Supplementary material for: Capillary leak syndrome due to snakebite in the Amazon: case report
Source: Rev Peru Med Exp Salud Publica. 2024 Oct 25;41(4):438–41. doi: 10.17843/rpmesp.2024.414.13614 (PMC11797586; doi:10.17843/rpmesp.2024.414.13614)
Supplement: Translation

# Translation: Síndrome de fuga capilar por mordedura de serpiente en la Amazonía. Reporte de un caso

**Authors:** Edgar A. Ramírez-García, Arley Perez-Mori, Mónica Mori-Coral, Maria Jose V. Canchanya-Olimar, Juan C. Celis-Salinas, Martín Casasapia-Morales

**Affiliations:** 1 Universidad Nacional de la Amazonia Peruana, Loreto, Perú.; 2 Hospital Regional de Loreto, Loreto, Perú.; 3 Sociedad Científica de Estudiantes de Medicina de la Amazonía Peruana, Loreto, Perú.

**Keywords:** Niño, Mordeduras de Serpientes, Síndrome de Fuga Capilar

## Abstract

El síndrome de fuga capilar es una complicación única de etiología variable que se caracteriza por la extravasación de líquidos en el espacio intersticial por la pérdida de proteínas a causa del envenenamiento por mordeduras de serpientes. Se describe el caso clínico de un niño de 12 años, natural del distrito de Napo en la ciudad de Iquitos en la Amazonía peruana, quien presentó edema y aumento del volumen del rostro por la mordedura de una serpiente del género *Bothrops* en cara lateral de la pierna derecha, posteriormente fue hospitalizado y se le diagnosticó de ofidismo grave complicado con edema de cara. El paciente recibió ocho viales de antiveneno, antibióticos y analgésicos. Finalmente, tras ocho días de hospitalización resultó en una evolución favorable y la recuperación siendo dado de alta.

## INTRODUCCIÓN

El envenenamiento por mordeduras de serpientes fue clasificado por la Organización Mundial de la Salud como una de las causas más importantes de mortalidad en todo el mundo, siendo las regiones más afectadas Asia sudeste, África subsahariana y Asia Meridional ^1^^,^^2^. A nivel mundial, cada año se registran entre 4,5 y 5,4 millones de casos por mordeduras de serpientes, unas 400,000 personas afectadas quedan discapacitadas de forma permanente y las muertes reportadas oscilan entre 81 000 y 138 000 ^3^^,^^4^. En la India, cada año unas 49 mil personas de zona rural fallecen a causa de envenenamiento por mordeduras de serpientes ^5^. Durante el 2023, en el Perú se han registrado un total de 461 casos de ofidismo siendo Loreto con 134, San Martín con 96 y Ucayali con 58 los departamentos con los mayores reportes a nivel nacional. Los grupos etarios más afectados son los adultos con 43,4% y de sexo masculino con 67,3% ^6^. Estimaciones recientes mencionan que las mordeduras de serpientes venenosas son causantes de múltiples discapacidades en los afectados, en su mayoría son adultos y jóvenes provenientes de países en vías de desarrollo con un inadecuado acceso a los servicios de salud, y que un número aún mayor de personas quedan con secuelas físicas y psicológicas permanentes ^7^^,^^8^. 

El síndrome de fuga capilar es una complicación única causada por mordeduras de serpientes de la familia *Viperidae* con una tasa de mortalidad del 58%, es caracterizada por tumefacción de las glándulas parótidas, quemosis, edema periorbitario junto a hipotensión, hipoalbuminemia y hemoconcentración a causa de la extravasación de líquidos, es una complicación mortal de etiología variable y de muy poca incidencia ^9^. Presentamos un caso probable de Síndrome de Fuga Capilar identificado en la Amazonía Peruana, que contribuye a visibilizar la importancia del diagnóstico y tratamiento oportuno para una adecuada evolución de la enfermedad, así como mostrar una manifestación poco frecuente de la enfermedad.

## REPORTE DE CASO

Paciente varón de 12 años, procedente de la comunidad de Santa Clotilde en el rio Napo ubicado a 1113 km de la ciudad de Iquitos en el distrito de Napo, Provincia de Maynas, región de Loreto. Sin antecedentes infecciosos de relevancia, el día uno (05/10/2022) fue mordido por una serpiente *Bothrops* (Jergón) mientras caminaba a orillas del rio Napo. Según la madre, la serpiente medía 50 cm aproximadamente, de cabeza triangular, de color marron, con manchas oscuras, de cola amarilla. A las tres horas de la mordedura, desarrolló dolor intenso y sangrado en la zona de la mordedura, cara lateral de la pierna derecha, por lo que recibió remedios caseros (un vaso con agua y sal y un segundo vaso con agua y azucar). Al día siguiente (06/10/2022), el dolor persistía, acompañado de inflamación en la pierna, por lo que se le administra penicilina intramuscular. En el tercer día (07/10/2022), persistia el dolor en la zona de la mordedura y el paciente presentó edema en la cara, motivo por el cual fue trasladado a un centro de salud donde se le administraron cuatro viales de suero antiofídico elaborados por el Instituto Nacional de Salud del Perú, dexametasona y clorfenamina. El cuarto día (08/10/2022) desarrollaba aumento del edema, y por persistencia del cuadro clínico es trasladado por vía aérea a la ciudad de Iquitos, llegando al Hospital Regional de Loreto el quinto día (09/10/2022), en la evaluación el paciente se encontraba hemodinámicamente estable, con signos vitales dentro de los rangos normales (Figura 1). En el examen físico, se observó palidez cutánea, edema facial y cervical, así como equimosis en la cara lateral de la pierna derecha (Figura 2).

**Figura 1:** Línea de tiempo de la enfermedad y evolución en paciente por mordedura de *Bothrops*

**Figura 2:** Equimosis en cara lateral de la pierna derecha por mordedura de *Bothrops*

Las glándulas parótidas en cara estaban notablemente inflamadas, lo que sugería una probable complicación secundaria al envenenamiento por *Bothrops* (Figura 3).

**Figura 3:** Síndrome de fuga capilar por ofidismo grave manifestado por edema e inflamación de las glándulas parótidas en la cara

En el hemograma inicial reveló anemia leve con un nivel de hemoglobina de 9,9 g/dL (valor normal ≥ 12,0 g/dL). El resto de los estudios complementarios, incluyendo el perfil de coagulación, se encontraron dentro de los parámetros normales. Se le dio tratamiento con hidratación intravenosa de cloruro de sodio al 0,9% y un régimen antibiótico con ceftriaxona (1,5 g cada 12 horas) y clindamicina (385 mg cada 8 horas), complementado con la administración de cuatro viales de suero antiofídico (Instituto Nacional de Salud) y metamizol (950 mg) para el control del dolor.

Luego de 7 días de tratamiento, el paciente mostró una mejora significativa, manteniéndose hemodinámicamente estable con una disminución notable del edema y del dolor. Finalmente, el (13/10/2022) fue dado de alta con indicaciones médicas para continuar el seguimiento ambulatorio.

## DISCUSIÓN

El presente caso es de importancia para la práctica médica en áreas donde las mordeduras por serpientes del género*Bothrops* son frecuentes, como la Amazonía peruana. Este tipo de envenenamiento genera un cuadro clínico complejo que puede presentar tanto manifestaciones locales como sistémicas. Este es un primer caso reportado en la Amazonía peruana de un probable síndrome de fuga capilar asociado a ofidismo. Los hallazgos clínicos, como el edema facial, sugieren la presencia de este síndrome, una rara complicación observada en envenenamientos por serpientes de la familia Viperidae, que produce alteraciones hemodinámicas caracterizadas por mialgias, sed excesiva, inflamación de las glándulas parótidas, quemosis conjuntival e hipotensión. Un aspecto crucial en la discusión de este caso es la posible presentación del síndrome de fuga capilar. Este trastorno, aunque poco común, es una complicación grave en los casos de envenenamiento por serpientes. Sin embargo, la literatura médica ha documentado la mayoría de los casos en estudios provenientes de la India, y su aparición en América del Sur es escasamente reportada. El síndrome de fuga capilar, también conocido como enfermedad de Clarkson, implica un aumento de la permeabilidad capilar, ocasionado por una elevada presión hidrostática en los capilares, lo que puede derivar en insuficiencia cardíaca, renal y obstrucción venosa hepática ^11^^,^^12^.

El síndrome de fuga capilar se caracteriza por la presencia de tumefacción parotídea bilateral, quemosis y edema periorbitario después de una mordedura de serpiente a partir del segundo o tercer día, la acumulación de líquido en la cara se ha denominado “aspecto de cabeza de víbora” por la inflamación de ambas glándulas parótidas ^13^. El envenenamiento por la víbora de Russell (Familia *Veperidae*) provoca una toxicidad vascular y hemorragia, también se ha observado una variación que puede conducir al síndrome de fuga capilar provocando edema generalizado y extravasación masiva de plasma, una condición causada por el aumento profundo de la permeabilidad vascular, lo que indica que el veneno del género *Viperidae* se asocia a esta complicación potencialmente mortal ^14^.

Para el diagnostico, se debe de evidenciar el aumento de la permeabilidad capilar por la pérdida de proteínas en el espacio intersticial con la aparición de fatiga, mareos, edema, hemoconcentración e hipoalbuminemia. Después de unos días, la permeabilidad mejora de forma espontánea y las funciones vitales se estabilizan. El manejo de fluidos es una parte esencial del tratamiento debido a que la hipovolemia y la hipotensión pueden empeorar el estado llevando a una falla multiorgánica ^15^. Sin embargo, el niño presentaba marcadores inflamatorios dentro de lo normal sin pérdida de proteínas siendo manejado con hidratación oportuna logrando su mejoría.

En conclusión, a pesar que los marcadores inflamatorios se encontraban dentro de lo normal, se encontraron características de ofidismo grave con síndrome de fuga capilar. La principal limitación de este caso es la falta de una confirmación diagnóstica del síndrome de fuga capilar. Sin embargo, la ausencia de fiebre, la hemoglobina disminuida sin evidencia de hemorragias y los recuentos de leucocitos bajos no mostraban relación con otras enfermedades. Los hallazgos clínicos de edema desde el ingreso junto con una marca de mordedura con equimosis en pierna derecha caracterizaron el envenenamiento por serpiente del género *Bothrops* (Familia Viperidae) que causa el probable síndrome de fuga capilar. El niño recibió ocho viales de suero antiofídico, de los cuales cuatro fueron en el centro de referencia y cuatro cuando llegó al hospital. Después de unos días de hospitalización, el paciente logró recuperarse siendo dado de alta al octavo día de hospitalización.
